# Loss of Fer Jeopardizes Metabolic Plasticity and Mitochondrial Homeostasis in Lung and Breast Carcinoma Cells

**DOI:** 10.3390/ijms22073387

**Published:** 2021-03-25

**Authors:** Linoy Mehazri, Sally Shpungin, Shai Bel, Uri Nir

**Affiliations:** 1 The Mina and Everard Goodman Faculty of Life-Sciences, Bar-Ilan University, Ramat-Gan 5290002, Israel; mahazrl@biu.ac.il (L.M.); Sally.Shpungin@mail.biu.ac.il (S.S.); 2 The Azrieli Faculty of Medicine, Bar-Ilan University, Safed 1311202, Israel; Shai.Bel@biu.ac.il

**Keywords:** Fer, non-small cell lung cancer, triple-negative breast cancer, metabolic plasticity, Mitochondrial homeostasis

## Abstract

Metabolic plasticity is a hallmark of the ability of metastatic cancer cells to survive under stressful conditions. The intracellular Fer kinase is a selective constituent of the reprogramed mitochondria and metabolic system of cancer cells. In the current work, we deciphered the modulatory roles of Fer in the reprogrammed metabolic systems of metastatic, lung (H358), non-small cell lung cancer (NSCLC), and breast (MDA-MB-231), triple-negative breast cancer (TNBC), carcinoma cells. We show that H358 cells devoid of Fer (H358ΔFer), strictly depend on glucose for their proliferation and growth, and fail to compensate for glucose withdrawal by oxidizing and metabolizing glutamine. Furthermore, glucose deficiency caused increased reactive oxygen species (ROS) production and induction of a DNA damage response (DDR), accompanied by the onset of apoptosis and attenuated cell-cycle progression. Analysis of mitochondrial function revealed impaired respiratory and electron transport chain (ETC) complex 1 (comp. I) activity in the Fer-deficient H358ΔFer cells. This was manifested by decreased levels of NAD^+^ and ATP and relatively low abundance of tricarboxylic acid (TCA) cycle metabolites. Impaired electron transport chain comp. I activity and dependence on glucose were also confirmed in Fer-deficient, MDA-MB-231ΔFer cells. Although both H358ΔFer and MDA-MB-231ΔFer cells showed a decreased aspartate level, this seemed to be compensated by the predominance of pyrimidines synthesis over the urea cycle progression. Notably, absence of Fer significantly impeded the growth of H358ΔFer and MDA-MB-231ΔFer xenografts in mice provided with a carb-deficient, ketogenic diet. Thus, Fer plays a key role in the sustention of metabolic plasticity of malignant cells. In compliance with this notion, targeting Fer attenuates the progression of H358 and MDA-MB-231 tumors, an effect that is potentiated by a glucose-restrictive diet.

## 1. Introduction

Metastasis is the main cause of death in cancer patients. Metastasis itself is a highly inefficient process, and experimental models suggest that only ~10% of intravenously injected cancer cells survive and extravasate, while most (>90%) of such cells die within 24 h of entry into the target organ [1]. It is assumed that during metastatic dissemination, cancer cells must alter their metabolism in a fashion that promotes survival and ultimately contributes to metastatic expansion [2]. Increasing evidence suggests that metastatic cells and primary tumor cells may utilize distinct metabolic pathways [3,4,5,6]. The aerobic glycolysis observed in primary tumors, a phenomenon known as the Warburg effect, is frequently replaced during metastasis by other routes of energy production. One of the main causes of this phenomenon may be the detachment of tumor cells from the extracellular matrix during metastasis, a process that impairs glucose uptake and extinguishes the Warburg effect. Consequently, this process enforces alternative metabolic adaptations, most commonly performed through mitochondrial metabolism [7,8]. Analysis of gene expression signatures in an orthotopic breast cancer model indicated that circulating tumor cells are enriched in factors that regulate mitochondrial respiration and biogenesis [3]. Accordingly, circulating tumor cells display increased levels of oxidative phosphorylation, mitochondrial biogenesis, and oxygen consumption [3]. These findings illustrate that metabolic plasticity is an integral metastatic trait.

The feline sarcoma-related (FER) proteins are members of a unique family of non-receptor tyrosine kinases. Members of this group are characterized by a kinase domain residing in the C-terminal region, which is preceded by an SH2 domain and a long N-terminal tail bearing three coiled-coil domains and an FCH (Fps/Fes/Fer/CIP4 Homology) motif [9,10,11].

The genomic *fer* locus is located on chromosome 5q21 [12], encoding the Fer kinase. Fer is expressed in all somatic cells, [13] except some immune cells [14] and resides in various subcellular compartments. However, only in spermatogenic and cancer cells does Fer associate with complex 1 (comp. I) of the mitochondrial electron transport chain (ETC) [15,16]. Thus, Fer is a cancer-specific component of the reprogrammed mitochondria of malignant cells. Notably, Fer has been shown to regulate breast cancer cell adhesion, migration and resistance to anoikis and to be necessary for tumor growth and metastases formation in mice [17]. Similarly, Fer has been shown to regulate migration and metastatic spreading of non-small cell lung cancer (NSCLC) and ovarian cancer cells [18,19]. Furthermore, high expression of Fer has been shown to serve as an independent prognostic factor that correlates with worse overall survival of triple-negative breast cancer (TNBC) and NSCLC patients [17,19,20,21].

Considering the fact that lung cancer was the deadliest form of cancer worldwide in 2020, and breast cancer was the first in incidence [22], we aimed to decipher the role of Fer in the development and progression of these cancer types.

In this study, we describe the importance of Fer in modulating the metabolic plasticity, mitochondrial homeostasis and tumor development capacity of metastatic bronchioloalveolar carcinoma (BAC)-NSCLC (H358) and TNBC (MDA-MB-231) cells. We also show that these findings may have translation ramifications.

## 2. Results

### 2.1. Impaired Metabolic Plasticity of Fer-Deficient H358 Cells

**Figure 1:**
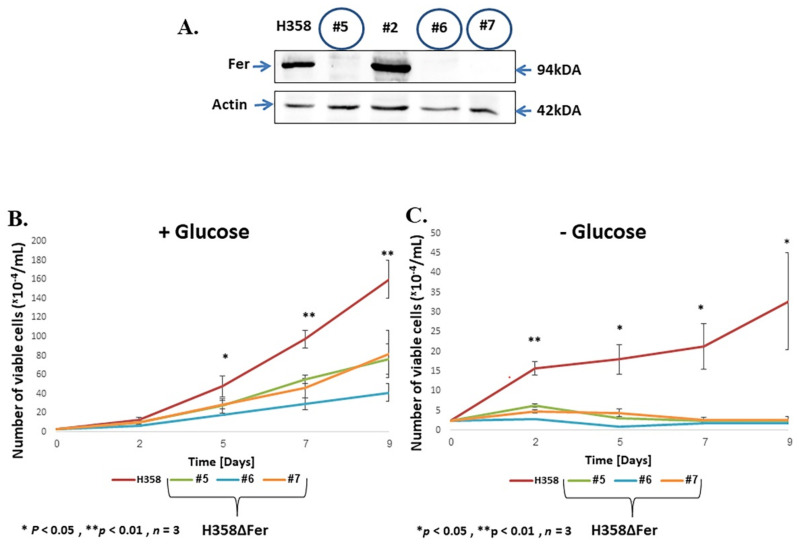

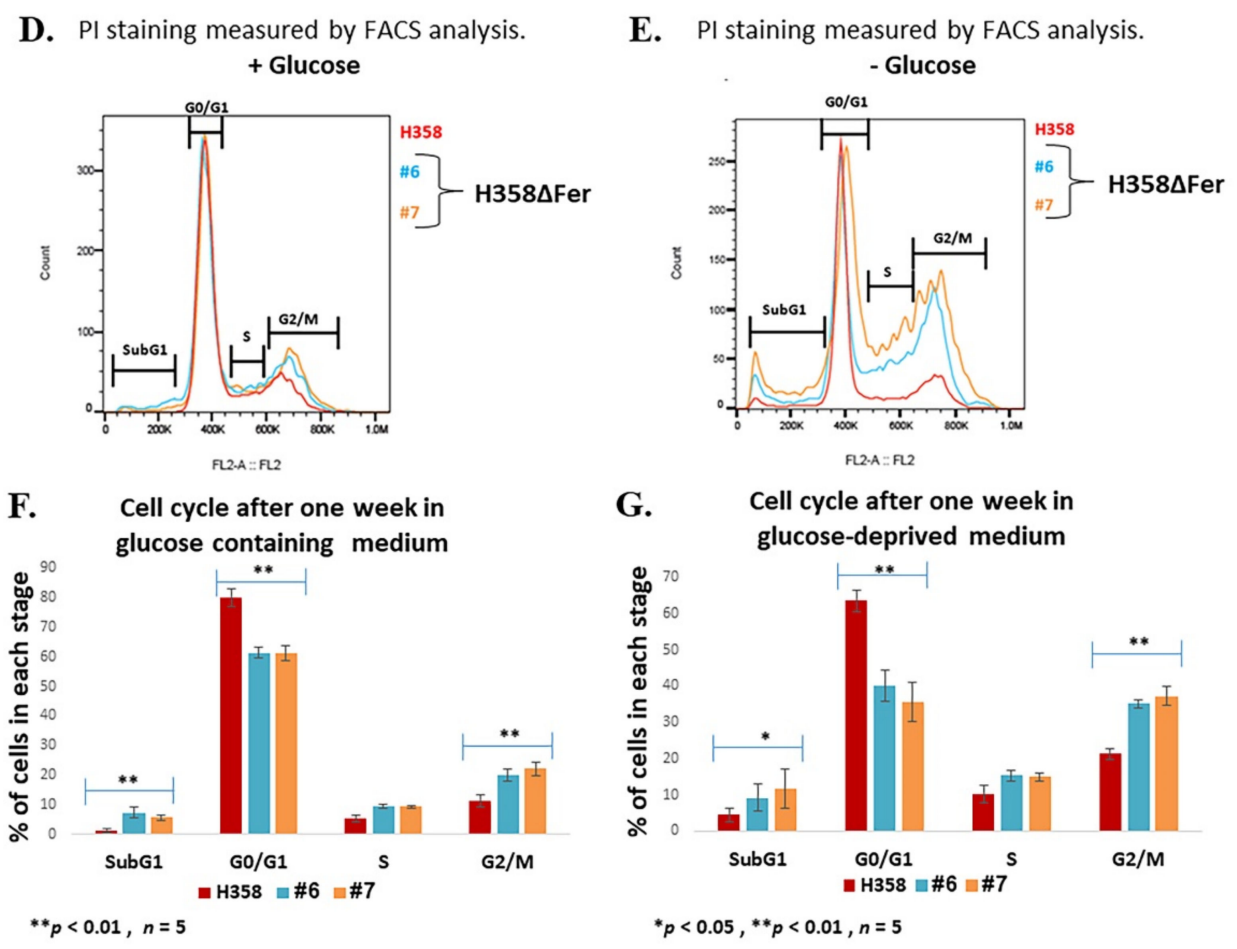
Impaired metabolic plasticity of Fer-deficient H358ΔFer cells. (**A**) Western blot analysis of protein lysates prepared from H358 variants. H358 denotes the parental cells and clones #5, #6 and #7 are CRISPR-Cas9-established H358ΔFer clones. Actin served as a loading control. (**B**) Growth curves of cells grown in a medium containing both glucose and glutamine or (**C**) in a glucose-free medium supplemented with glutamine. Values shown represent means +/− standard error (SE) (*n* = 3). (**D**) Propidium iodide (PI) staining and flow-cytometry cell-cycle analysis of cells grown in a medium supplemented with glucose and glutamine or (**E**) with glutamine alone. (**F**,**G**) Quantification of the results obtained in five independent experiments (**D**,**E**), respectively. Histograms represent means +/− SE (*n* = 5). ** *p*-value < 0.01.

To decipher the functions of Fer in lung cancer cells, we focused on the metastatic BAC-NSCLC H358 cell line [23,24], which highly expresses Fer (Figure 1A). We generated Fer-deficient H358 clones (H358ΔFer) by applying the modified CRISPR-Cas9 mutated knock-out system [25,26]. Expression of Fer was analyzed using western blot (WB) analysis, revealing efficient knockout of the *fer* locus in clones #5, #6 and #7, which fail to express the Fer protein (Figure 1A).

Since Fer associates with the mitochondrial comp. I in malignant cells [16], we sought to examine the effect of Fer deficiency on the growth profile of the H358ΔFer cells under different metabolic growth conditions. H358ΔFer clones and their parental H358 cells were seeded in RPMI growth medium containing 2 g/L glucose to propel glycolysis and 0.3 g/L glutamine to drive the progression of the mitochondrial tricarboxylic acid (TCA) cycle and oxidative phosphorylation processes [27,28]. Comparison of the growth curves of the two cell types revealed attenuated growth of the H358ΔFer clones compared to the parental H358 cells when grown with glucose-supplemented medium (Figure 1B). This effect was exacerbated upon growth of the H358ΔFer cells in a glucose-deprived and glutamine-supplemented medium (Figure 1C), conditions under which mitochondrial activity prevails as the main energy source. Thus, while the presence of Fer endows H358 cells with metabolic plasticity, which enables them to maintain growth under glucose deprivation, Fer-deficient cells seem to lose this resilience.

To reveal what could cause the attenuated growth of the Fer-deficient cells, we compared the cell-cycle progression profiles of the parental-H358 and H358ΔFer cells. Flow-cytometry analysis revealed attenuated progression of the cell-cycle G2/M transition stage in H358ΔFer cells grown in a glucose- and glutamine-supplemented medium (Figure 1D,F). This was reflected by an approximately 50% increase in the number of cells residing in the G2/M stage during the cell-cycle progression, and an accompanying reduction in the percentage of the G0/G1-residing H358ΔFer cells. A marginal increase in the percentage of sub-G1 H358ΔFer cells indicated an elevation in cellular death level in the absence of the Fer protein (Figure 1D,F). The increase in the relative number of G2/M and sub-G1 H358ΔFer cells was exacerbated when the cells were grown in the absence of glucose and with glutamine as their major energy source (Figure 1E,G). These results suggest that attenuated G2/M progression and onset of cellular death, contribute to the impaired growth of H358ΔFer cells both in the presence of glucose, and more profoundly, in its absence.

### 2.2. Elevated ROS Levels, Activation of the DNA Damage Response and Jeopardized Survival of Metabolically Challenged H358ΔFer Cells

**Figure 2:**
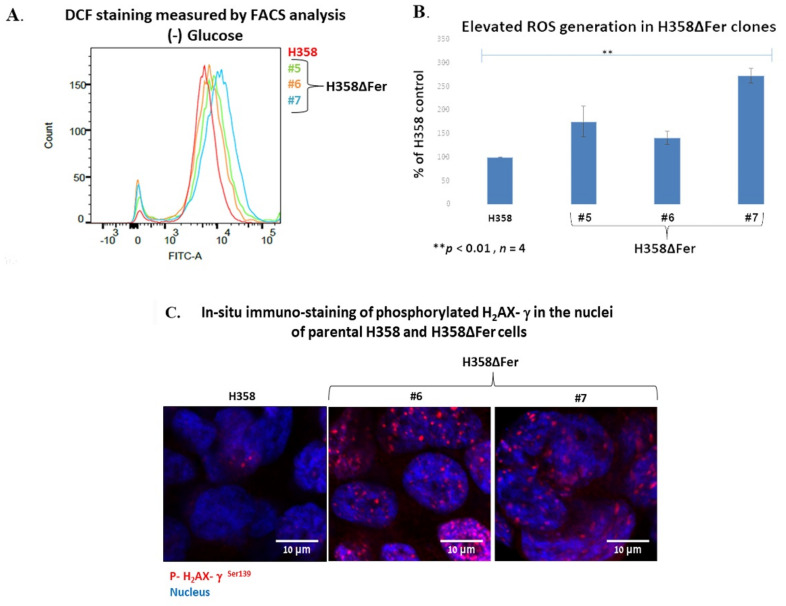

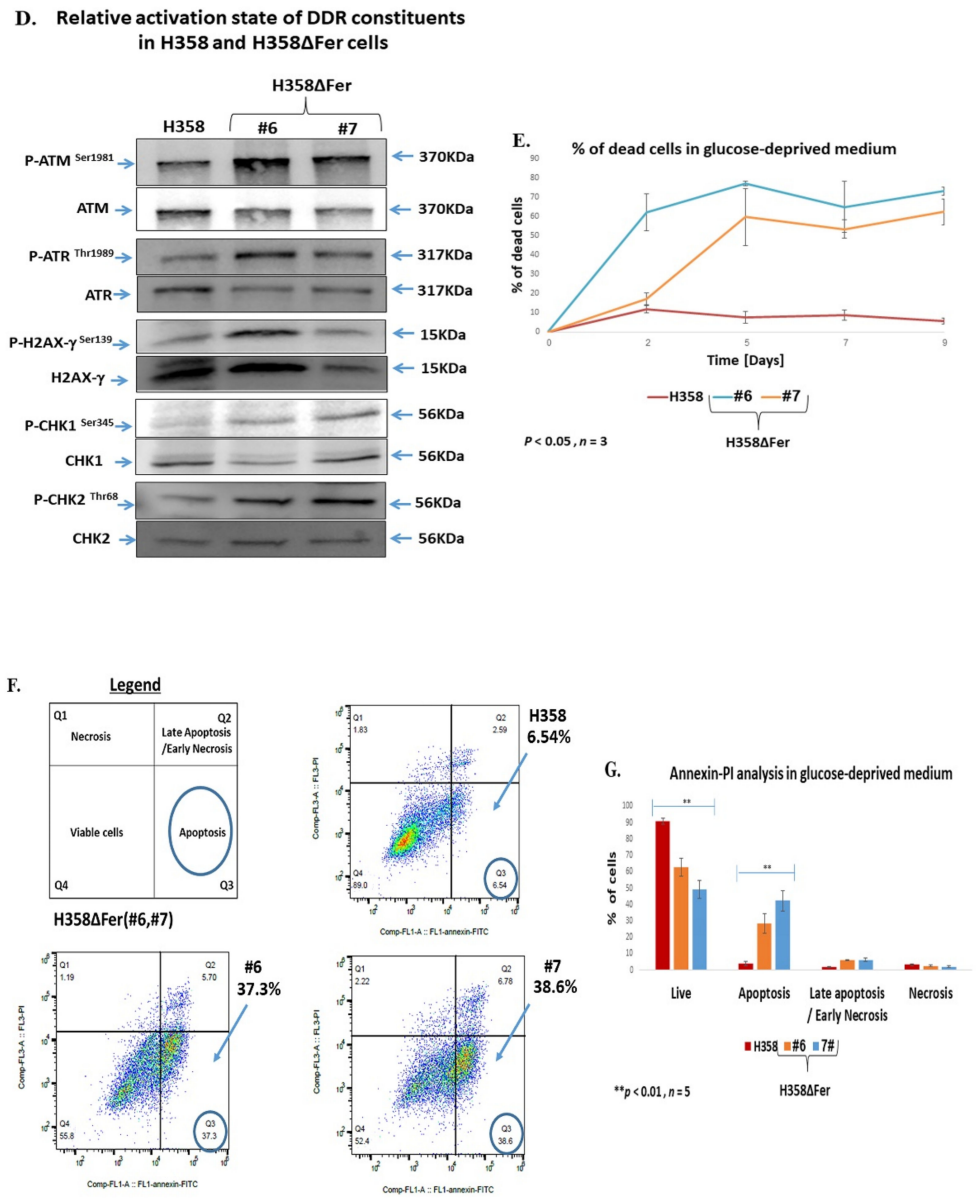
Elevated reactive oxygen species (ROS) levels, activation of DNA damage response (DDR), and onset of death, in metabolically challenged H358ΔFer cells. (**A**) Dichlorofluorescein (DCF) staining indicating ROS levels determined using flow-cytometry analysis of cells grown under glucose-deprivation and glutamine-supplementation conditions. Profiles show one representative experiment out of four independent experiments that gave similar results. (**B**) Quantification of four independent ROS-determining experiments; results are presented as percent difference between H358ΔFer and H358 cells. (**C**) In-situ immuno-staining of phosphorylated H_2_AX-γ (red) in the nuclei (blue) of parental H358 and H358ΔFer cells. Scale bar: 10 µm. (**D**) WB analysis depicting the relative activation state of DDR constituents in H358 and H358ΔFer cells supplemented with glutamine in the absence of glucose. Results shown represent one out of three independent experiments which gave similar results. (**E**) Death curves of cells grown in glucose-free medium supplemented with glutamine was determined using the Trypan blue uptake-assay (**F**) Annexin-PI staining and FACS analysis determining the viability of cells grown under glucose deprivation and glutamine supplementation. Images represent one out of five independent experiments which gave similar results. (**G**) Average values were obtained from all five independent experiments. Results are presented as percentage of cells in each subpopulation. Histograms represent means +/− SE. ** *p*-value < 0.01.

Knockdown of Fer was shown to increase the accumulation of reactive oxygen species (ROS) in malignant cells [16]. We therefore determined the ROS levels in the parental and Fer-deficient H358ΔFer cells, when grown under glucose deprivation. ROS levels were significantly increased in H358ΔFer cells when compared to the parental H358 cells (Figure 2A,B).

Elevated levels of ROS can cause nuclear DNA damage and consequent activation of the DNA damage response (DDR), which by itself can lead to G2/M arrest and attenuation of the cell-cycle progression [29]. We therefore tested whether growth of H358ΔFer cells under glucose deprivation evokes DNA damage. Cells were immuno-stained for the phosphorylated form of the histone variant, H_2_AX-γ, in the nuclei of the parental and H358ΔFer cells. This histone variant becomes activated through phosphorylation and is recruited to sites of DNA damage [30], thereby reflecting the onset of DNA damage in the DNA. The in-situ immuno-staining revealed increased accumulation of phosphorylated H_2_AX-γ in the nuclei of H358ΔFer cells when compared to the parental H358 cells (Figure 2C). This was corroborated by a WB analysis, which demonstrated the increased phosphorylation of H_2_AX-γ in H358ΔFer cells (Figure 2D). The onset of DNA damage induces the activation of a set of DDR cascades that enable cells to contend with the evoked damage. The serine/threonine kinases, ATM and ATR, are central components of these pathways and activate numerous downstream effectors, including the CHK1 and CHK2 kinases, which can attenuate the progression of the G2/M transition phase of the cell cycle [31,32,33,34]. To compare the relative activation state of the DDR constituents in the parental versus the Fer-deficient H358ΔFer cells, we examined the relative phosphorylation levels of ATM, ATR, CHK1 and CHK2 in the parental H358 and H358ΔFer cells. This analysis revealed the relative upregulation and induced phosphorylation of all four proteins in the H358ΔFer clones compared to the parental cells (Figure 2D). Figure S1A–E present the quantification histograms of phospho/pan ratios of the proteins presented and indicated in Figure 2D.

In addition, high doses of ROS can increase the permeability of the outer mitochondrial membrane and trigger apoptosis [35]. Thus, we sought to compare cell death levels in the presence or absence of Fer in cells grown under glucose deprivation. While the Fer-deficient cells showed increased death over time, the parental H358 cells showed a low percentage of cell death (Figure 2E). To characterize the type of death evoked in H358ΔFer cells, an Annexin/propidium iodide (PI) analysis was performed on the two cell types. This revealed that Fer depletion mostly triggered apoptotic death in the H358ΔFer cells rather than late apoptosis (Figure 2F,G), which by itself is represented by the sub-G1 population in the flow-cytometry cell-cycle analysis (Figure 1G) [36]. This distinction may explain the relatively modest increase of the sub-G1 population in glucose deprived H358ΔFer cells.

### 2.3. Impaired Mitochondrial Function in Fer-Deficient H358 Cells

**Figure 3:**
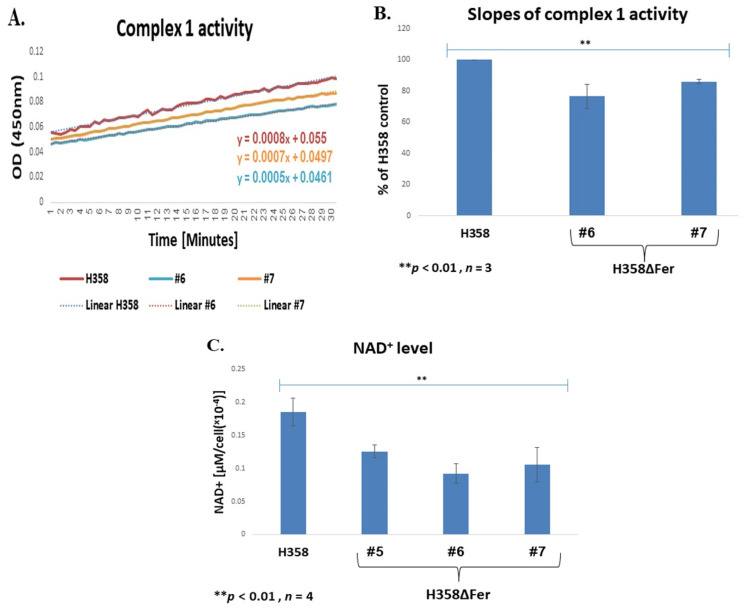

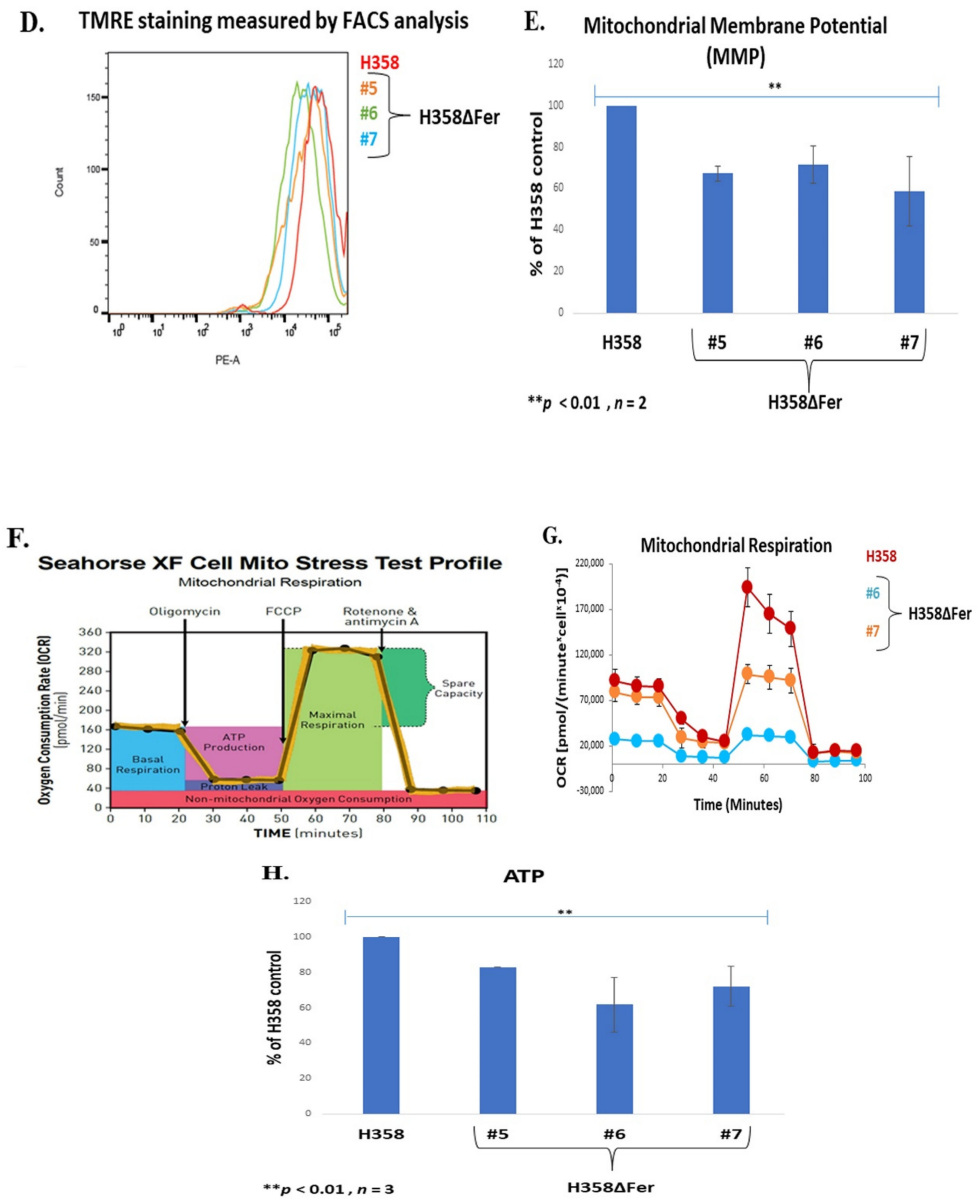
Impaired mitochondrial function in Fer-deficient H358ΔFer cells. (**A**) Impaired comp. I activity in H358ΔFer cells. Mitochondrial OXPHOS comp. I (NADH dehydrogenase) enzymatic activity. Results are presented as absorbance at OD 450 nm over time, and (**B**) reaction rates are presented as the slopes. (**C**) Reverse HPLC analysis of the NAD^+^ levels in H358 and H358ΔFer cells. These results represent one out of four independent experiments which gave similar results. (**D**) TMRE staining analysis measuring MMP, performed on H358 cells and H358ΔFer clones #5, #6 and #7. TMRE staining intensity determined by FACS analysis. (**E**) Quantification of the results in (**D**), presented as % of the parental H358, Fer-expressing cells. (**F**,**G**) Mito-stress analysis evaluating mitochondrial respiratory capacity in H358 cells and two H358ΔFer clones. (**G**) Shown is one out of three independent experiments which gave similar results. (**H**) LC-MS analysis of the ATP levels in H358 cells and H358ΔFer clones. Results are presented as % difference from the H358 Fer parental cells. Histograms represent means +/− SE. ** *p*-value < 0.01.

Increased ROS production can coincide with impaired activity of comp. I [37,38]. To examine whether comp. I activity is affected in H358ΔFer cells, we compared the comp. I activities in the parental H358 and the Fer-deficient H358ΔFer cells. This comparative analysis showed about a 20% decrease in comp. I activity in H358ΔFer cells (Figure 3A,B). The impairment in comp. I activity was also reflected by the decrease in the NAD^+^ levels [39] measured in the H358ΔFer clones (Figure 3C). In addition, H358ΔFer cells showed significantly reduced tetramethylrhodamine, ethyl ester (TMRE) staining compared to the parental H358 cells, an indication of a decreased mitochondrial membrane potential in the Fer-deficient cells (Figure 3D,E).

To further compare the mitochondrial activity of the different cell-types, we analyzed the oxygen-consumption rate of the parental and Fer-deficient H358ΔFer clones. This examination revealed decreased mitochondrial respiration capacity in the absence of Fer (Figure 3F,G), accompanied by a decreased ATP level in the H358ΔFer cells (Figure 3H). These findings imply that absence of Fer impairs mitochondrial function in Fer-deficient cells.

### 2.4. Fer Sustains Mitochondrial Metabolic Homeostasis in H358 and MDA-MB-231 Cells

**Figure 4:**
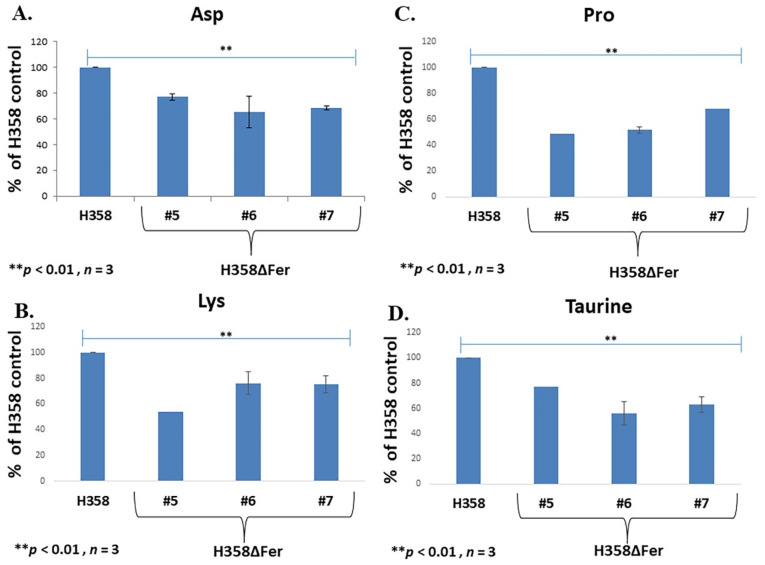
Fer sustains mitochondrial homeostasis in H358 cells. (**A**) Reverse HPLC analysis of the TCA metabolite- aspartate, levels, (**B**) LC-MS analysis of Lys, (**C**) Pro and (**D**) methionine-derived taurine amino acids in H358 cells and H358ΔFer clones. Cells were supplemented with glutamine and grown in the absence of glucose. Histograms represent means +/− SE (*n* = 3). ** *p*-value < 0.01**.**

The central role of the ETC in proliferative cells is to oxidize NADH for the generation of the non-essential amino acid, aspartate [40]. As we detected decreased NAD^+^ levels in H358ΔFer cells (Figure 3C), we also sought to measure the cellular aspartate levels before and after Fer deficiency. Reverse high pressure/performance liquid chromatography (HPLC) analysis revealed a lower level of aspartate in the H358ΔFer clones compared to the parental cells (Figure 4A) In addition, liquid chromatography–mass spectrometry (LC-MS) analysis revealed that H358ΔFer cells show decreased levels of Lys, Pro, and the methionine-derived taurine amino acid (aa); all of these are derived metabolic intermediates of the TCA cycle (Figure 4B–D). Thus, in the absence Fer, the mitochondrial metabolic homeostasis was being distorted.

**Figure 5:**
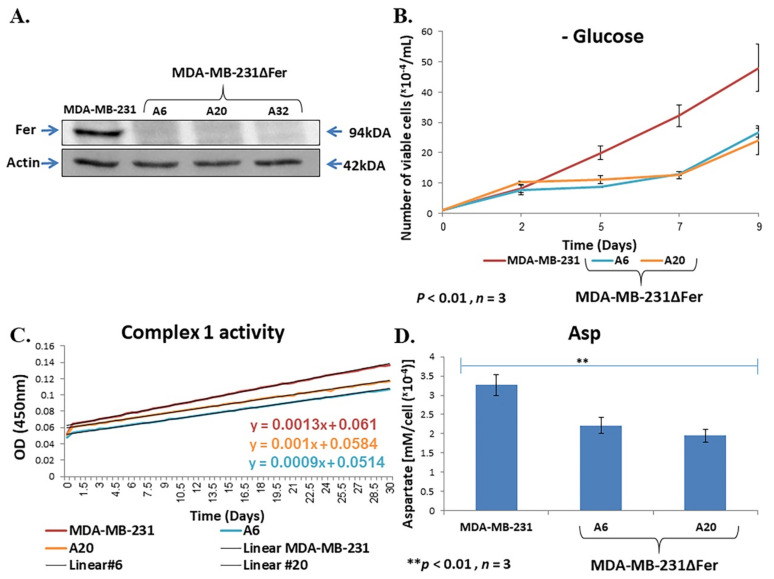
Fer sustains metabolic plasticity and mitochondrial homeostasis in MDA-MB-231 cells. (**A**) WB analysis of Fer level in MDA-MB-231 cells and in Fer-deficient MDA-MB-231ΔFer clones, A6, A20 and A32. Actin served as a loading control. (**B**) Growth curves of MDA-MB-231 and MDA-MB-231ΔFer cells grown in glucose-free medium, supplemented with glutamine. Values shown represent means +/− SE (*n* = 3). (**C**) Impaired comp. I activity in MDA-MB-231ΔFer cells. Mitochondrial OXPHOS comp. I (NADH dehydrogenase) enzymatic activity in the parental and the Fer-deficient clones. Results are presented as absorbance at OD 450 nm over time. These results represent one out of three independent experiments which gave similar results. (**D**) Reverse HPLC analysis of aspartate levels in MDA-MB-231 and MDA-MB-231ΔFer cells. Histograms represent means +/− SE (*n* = 3). ** *p*-value < 0.01.

To check the generality of our findings, we applied a CRSPR-Cas9 strategy to selectively knock-out the *fer* gene in another metastatic cancer cell-line. Since similarly to NSCLC [19,20,21], Fer has also been linked to poor prognosis in TNBC patients [17], we chose the metastatic TNBC-MDA-MB-231 cell line [41], which also highly expresses Fer (Figure 5A). The obtained clones were analyzed, and of the three obtained Fer-deficient MDA-MB-231ΔFer clones, two were taken for further analysis (Figure 5A). As seen with H358ΔFer cells, Fer-deficient MDA-MB-231 (MDA-MB-231ΔFer) cells showed attenuated growth under glucose deprivation conditions (Figure 5B), impaired comp. I activity (Figure 5C) and decreased aspartate level (Figure 5D) compared to the parental MDA-MB-231 cells. These results corroborate our notion that Fer supports metabolic plasticity and mitochondrial function and homeostasis in malignant cells.

### 2.5. Down-Regulated Urea Cycle in Fer-Deficient H358ΔFer and MDA-MB-231ΔFer Cells

**Figure 6:**
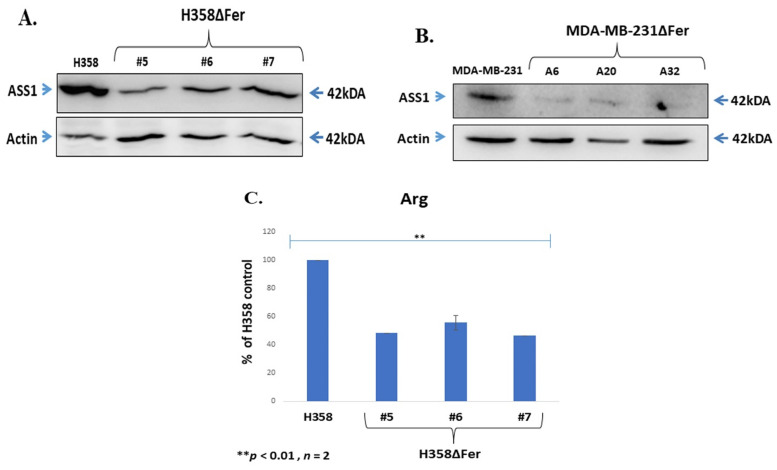

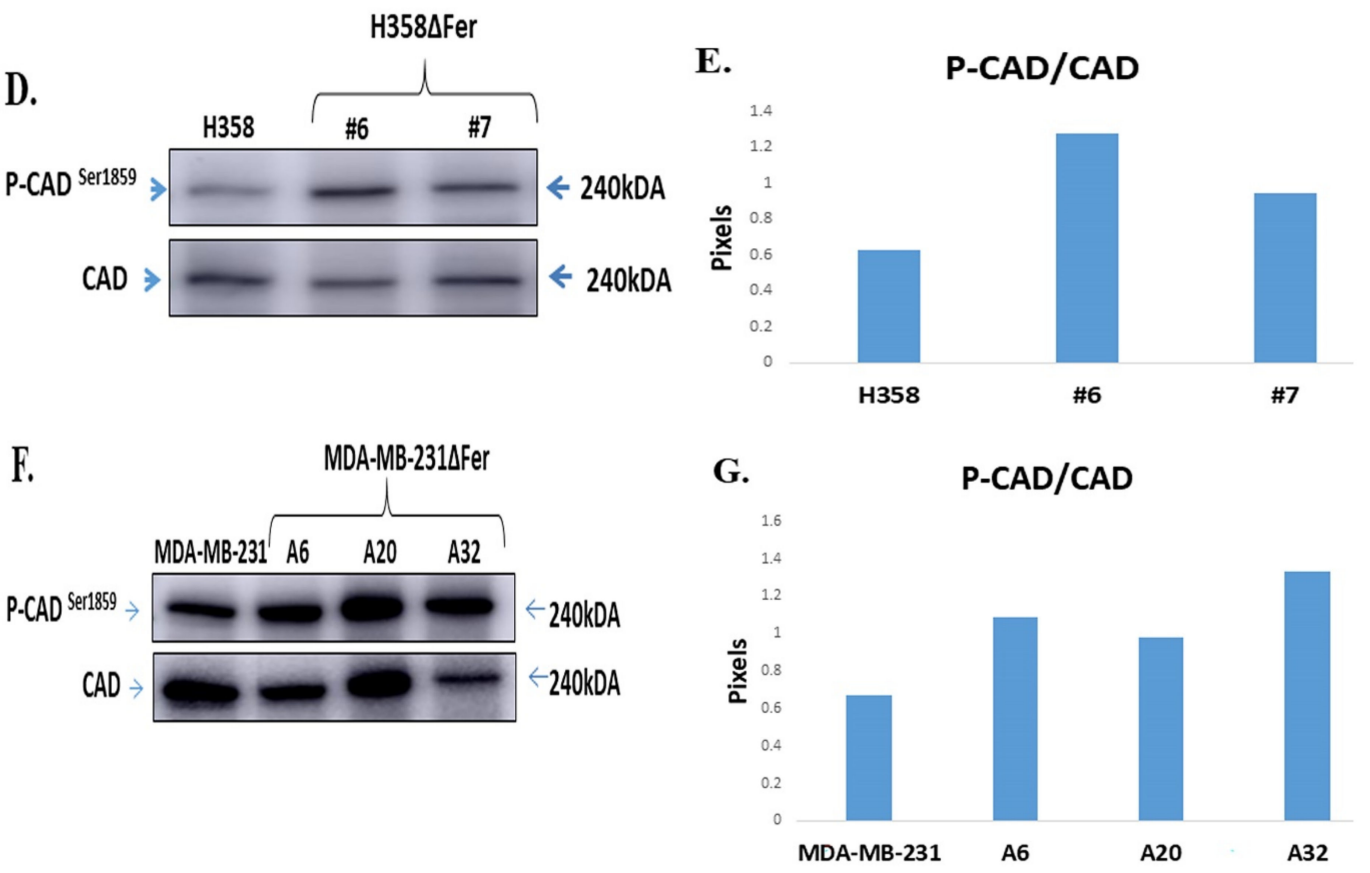
Down-regulated urea cycle in Fer-deficient H358ΔFer and MDA-MB-231ΔFer cells. (**A**) WB analysis depicting the relative expression levels of Argininosuccinate Synthase 1 (ASS1) in H358 and H358ΔFer cells and (**B**) in MDA-MB-231 and MDA-MB-231ΔFer cells. Actin served as a loading control. (**C**) Decreased arginine levels in H358ΔFer cells grown in the absence of glucose. Histograms represent means +/− SE of 2 independent experiments (** *p*-values < 0.01). (**D**,**E**) WB analysis depicting the relative expression levels of phospho- carbamoyl-phosphate synthetase 2, aspartate transcarbamylase, and dihydrooroatase (p-CAD) in H358 and H358ΔFer and (**F**,**G**) in MDA-MB-231 and MDA-MB-231ΔFer cells. CAD pan levels served as a loading control. (**E**,**G**) Quantification of the results presented in (**D**,**F**), respectively. These results represent one out of two independent experiments which gave similar results.

Aspartate serves as a key component of the pyrimidine-synthesis process [42]. Decreased aspartate levels in Fer-deficient cells (Figure 4A and Figure 5D) were therefore expected to lead to decreased cellular levels of pyrimidines and consequent attenuated DNA replication processes in Fer-deficient cells [43]. Since we did not see a significant effect of Fer deficiency on the cell-cycle S-phase progression, we determined the pyrimidine levels in Fer-proficient and deficient cells. LC-MS analysis results showed similar pyrimidine levels in H358ΔFer clones, compared to the parental H358 cells (Figure S2A–C). A previous study has linked impaired aspartate availability to the downregulation of arginosuccinate synthase (ASS1), thereby enabling the direction of aspartate away from the urea cycle toward pyrimidines biosynthesis [44]. Argininosuccinate Synthase 1 (ASS1) is a rate-limiting enzyme in urea synthesis and its down-regulation allows prevailing oxidative consumption in the pyrimidines synesthetic pathway [44,45]. To examine the ASS1 levels in parental and Fer-deficient cells, we performed WB analysis which revealed decreased ASS1 expression levels in the absence of Fer in both H358ΔFer and MDA-MB-231ΔFer cells (Figure 6A,B). Since ASS1 catalyzes the penultimate step of the arginine biosynthetic pathway in the urea cycle [46], we decided to further determine the relative arginine levels, marking the processing of the urea cycle. LC-MS analysis of H358ΔFer cells revealed a decreased arginine level compared to the parental H358 cells (Figure 6C).

Since both H358ΔFer and MDA-MB-231ΔFer cells showed decreased ASS1 expression levels, we proceeded to examine whether this decrease was linked to a diversion of the available aspartate towards pyrimidine synthesis. While the urea cycle is propelled by ASS1 activity [45], *de novo* biosynthesis of pyrimidines is controlled by the carbamoyl-phosphate synthetase 2, aspartate transcarbamylase, and dihydrooroatase (CAD) enzyme, which is activated through phosphorylation [44,47]. Indeed, WB analysis showed increased phosphorylation levels of CAD on Ser^1859^ in both H358ΔFer and MDA-MB-231ΔFer clones (Figure 6D–G), indicating an elevated CAD activity in the Fer-deficient cells.

These findings indicate that the decreased level and metabolic availability of aspartate, is compensated by its favored direction toward pyrimidines synthesis, in the Fer-deficient cells.

### 2.6. Absence of Fer Impedes the Development of H358 and MDA-MB-231 Xenografts in Mice

**Figure 7:**
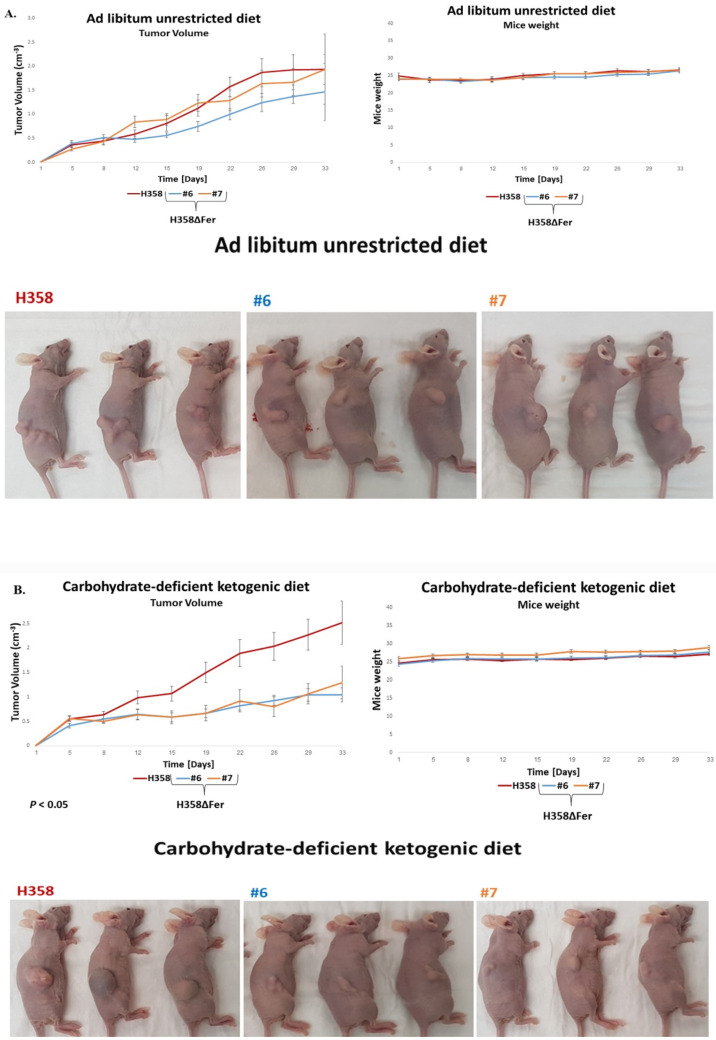

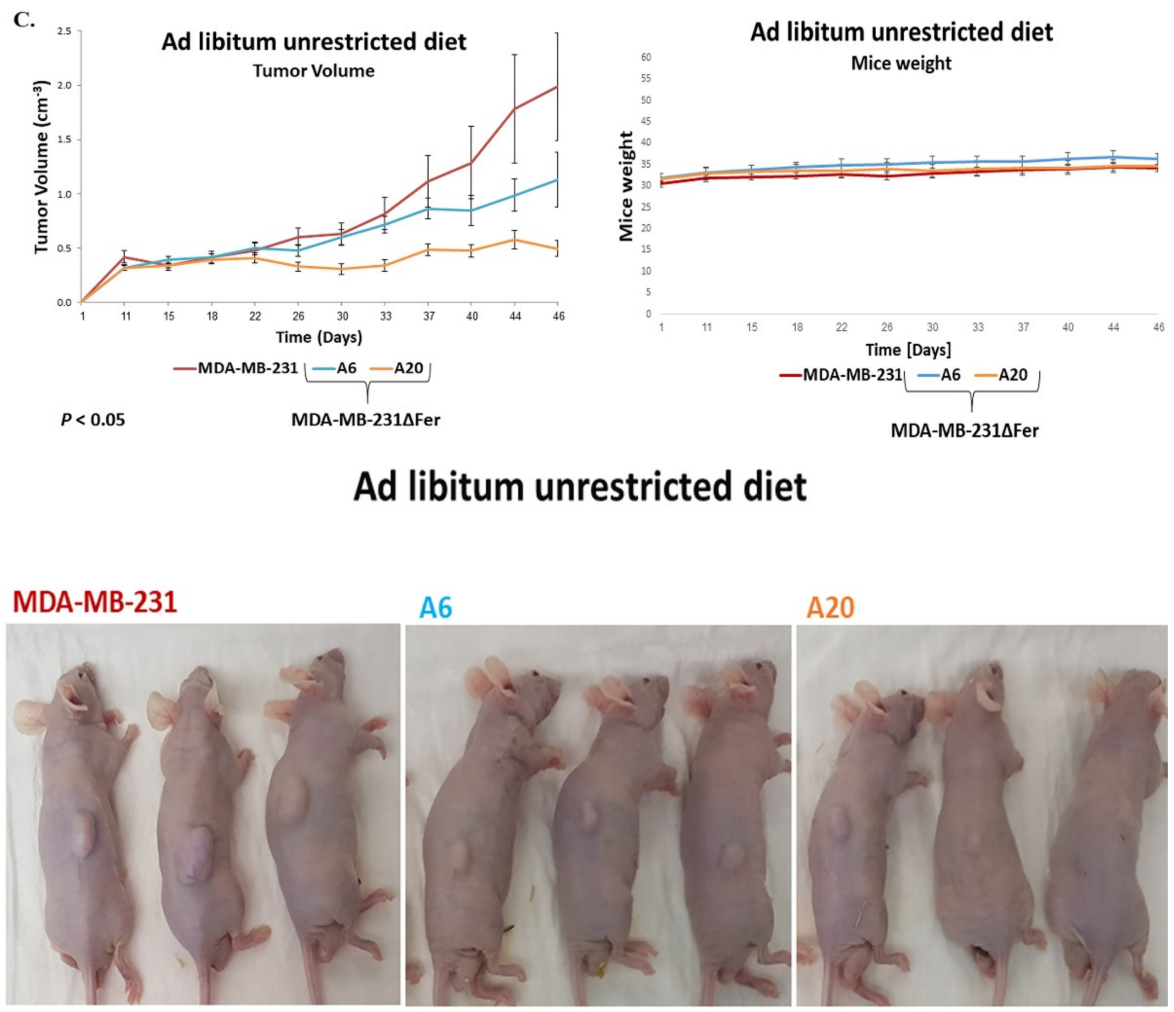

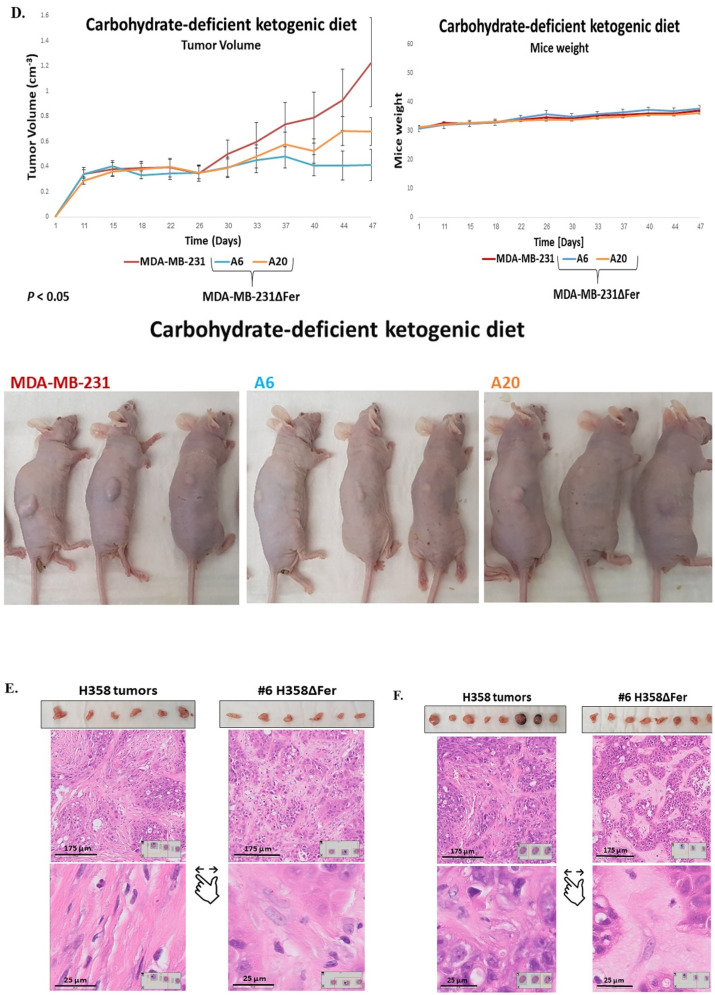

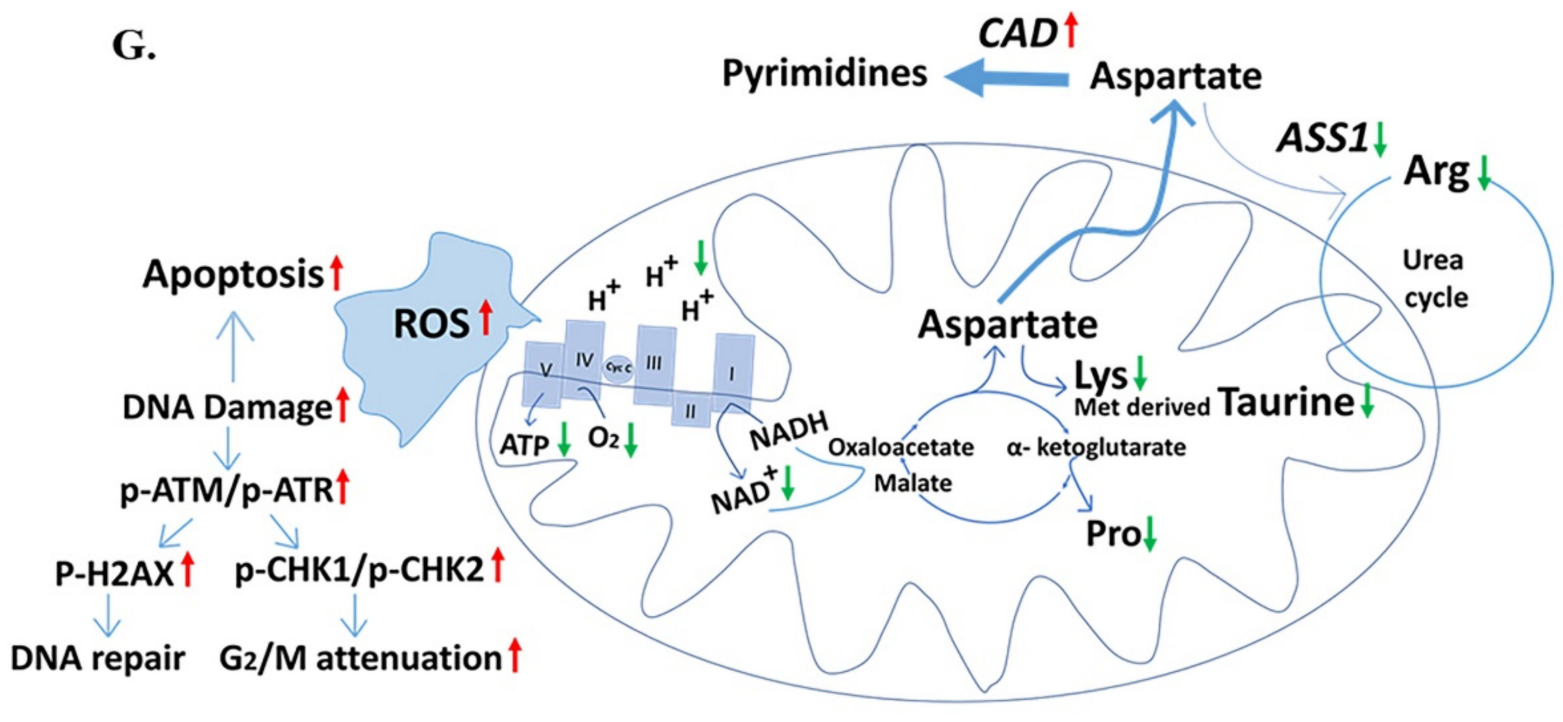
Absence of Fer impedes the development of BAC-NSCLC H358 and TNBC MDA-MB-231 xenografts in mice. Tumor-growth profiles, body-weight graphs of mice, and representative pictures of mice carrying tumors of (**A**,**B**) H358 and H358ΔFer (clones #6, #7) cells and (**C**,**D**) MDA-MB-231 and MDA-MB-231ΔFer (clones A6, A20, A32) cells, in mice fed with (**A**,**C**) a regular ad libitum unrestricted diet, or (**B**,**D**) a carbohydrate-deficient ketogenic diet. Values shown represent means +/- SE (*n* = 8). (**E**) Hematoxylin and eosin staining of H358 and H358ΔFer sections derived from tumors in mice fed with an ad libitum unrestricted diet, or (**F**) a carbohydrate-deficient ketogenic diet. Scale bar: 200 µm. (**G**) Summarizing scheme depicting the supportive role of Fer in sustaining the metabolic plasticity of H358 NSCLC and MDA-MB-231 TNBC cells. Shown are mitochondrial enzymes and metabolites which are upregulated (red arrows) or downregulated (green arrows) in Fer-deficient cells.

After deciphering the metabolic regulatory roles of Fer in-vitro, we intended to test the implications of these roles on the growth of xenografts derived from the different H358 and MDA-MB-231 cell variants, in-vivo. Since absence of Fer caused the H358ΔFer and MDA-MB-231ΔFer cells to become dependent on glucose for growth (Figure 1B,C and Figure 5B), we aimed to examine and compare the effect of Fer deletion on the formation of tumors in mice fed with either an ad libitum unrestricted diet, or with a carbohydrate-deficient ketogenic diet. To this end, H358 and MDA-MB-231 cells, as well as their Fer-deficient derived clones (H358ΔFer and MDA-MB-231ΔFer respectively), were subcutaneously injected into immuno-compromised (nude) mice, and the development and progression of the elicited tumors were followed. Notably, while the progression of H358ΔFer xenografts was only marginally attenuated (an average of 12% decrease in tumor’s volume) in comparison to the H358 xenografts in mice fed with an ad libitum diet (Figure 7A), under ketogenic diet this difference reached 46% (Figure 7B). Similarly, while an ad libitum diet impeded the development of MDA-MB-231ΔFer xenografts, a carb-deficient ketogenic diet halted the MDA-MB-231ΔFer tumors progression (Figure 7C,D).

To examine whether the attenuated growth of the H358ΔFer tumors could be reflected by their altered histological structure, we performed hematoxylin and eosin staining of tumors’ cross sections. The most prominent difference seen was in the tissue density of the different tumors. While sections of the parental H358 and H358ΔFer tumors removed from ad libitum fed mice looked similar and highly dense (Figure 7E), the sections of Fer deficient H358ΔFer xenografts removed from mice fed with a carb-deficient ketogenic diet were watery and significantly less dense compared to sections of the parental H358 tumors removed from similarly fed mice (Figure 7F). Similar patterns were seen in sections prepared from MDA-MB-231 and MDA-MB-231ΔFer xenografts (Figure S3A,B).

Thus, the outcome of jeopardized metabolic plasticity of Fer-deficient tumors challenged with restricted glucose availability can also be seen in their altered and distorted texture.

## 3. Discussion

Metastatic cells require high metabolic plasticity, adaptability and metabolic-stress resistance [1,2,3,4,5,6]. Furthermore, the disengagement in cancer cells between aerobic glycolysis, which is shunted toward lactic acid production [48], and mitochondrial oxidative phosphorylation necessitates the reprogramming of metabolic mitochondrial pathways in malignant cells. For example, the mitochondrial glutamine uptake machinery, which propels the TCA cycle, is upregulated in cancer cells [27,28]. Notably, other functional pathways, including the ETC, were found to be altered and mutated in the mitochondria of malignant cells. Specifically, comp. I mutations which could underlie the sensitivity of malignant cells to glucose limitation are frequently observed in many cancers [49].

In the current study, we further examined the functional role of another alteration in the ETC of cancer cells, namely, the association of the intracellular tyrosine kinase Fer with comp. I, which occurs in malignant but not in normal somatic cells [15,16]. Absence of Fer in lung (H358ΔFer cells) and breast (MDA-MB-231ΔFer) carcinoma cells, jeopardized their metabolic plasticity and disabled their growth under glucose-deprivation conditions**,** which enforced their reliance on mitochondrial metabolism. The impaired growth of H358ΔFer cells under glucose deprivation reflected their attenuated cell-cycle progression due to an impaired G2/M transition and the increased apoptotic death evoked under the imposed metabolic stress. Both the cell-cycle arrest and apoptosis could be attributed to the induction of DDR, which by itself, could be activated by increased production of ROS in the H358ΔFer cells [29]. Absence of Fer impaired the comp. I activity and mitochondrial functioning in the H358ΔFer cells, an effect that leads to the enhanced production of ROS [37,38,50] and, consequently, to the induction of DDR. The requirement of Fer for ensuring proper activity of comp. I and mitochondrial function in malignant cells reflects the reprograming of the ETC in the mitochondria of cancer cells. This reprogramming might endow malignant cells with metabolic plasticity, which is of profound importance for the survival and proliferation of metastatic cells. Impaired comp. I activity in Fer-deficient cancer cells could have further metabolic ramifications. It has been shown that the mitochondrial ETC enables the progression of numerous TCA-linked metabolic processes [40,51]. Of these metabolic processes, the progression of aspartate synthesis, which depends on the integrity of the ETC, should be noted. Moreover, it has been demonstrated that supporting aspartate synthesis is an essential function of respiration in proliferating cells [40,52]. In compliance with this notion, Fer-deficient H358ΔFer and MDA-MB-231ΔFer cells showed decreased aspartate levels, accompanied by reduced levels of other TCA intermediate metabolites like the aa, Lys, Pro and the methionine-derived taurine [53]. The shortage in aspartate, which can lead to deficiency in pyrimidines [52], was compensated in both H358ΔFer and MDA-MB-231ΔFer cells through down-regulation of the urea-cycle rate-limiting enzyme, ASS1 [46]. Down-regulation of the urea-cycle allowed, through activation and upregulation of the CAD enzyme, a preferred diversion of aspartate toward pyrimidines synthesis [44]. In spite of these reprogrammed, compensatory metabolic pathways, the dependence of the Fer-deficient cancer cells on glucose, and their jeopardized metabolic plasticity, impacted their proliferation and growth, not only in-vitro, but in-vivo as well.

This was reflected by the effect of a ketogenic diet on the development and progression of H358ΔFer and MDA-MB-231ΔFer tumors in immunocompromised mice. While the progression rate of the H358ΔFer xenografts in mice fed with an unrestricted ad libitum diet was attenuated in comparison to that of the parental H358 tumors, this attenuation was significantly exacerbated in mice fed with a carb-deficient ketogenic diet. Notably, the progression of MDA-MB-231ΔFer xenografts was also significantly attenuated under ad libitum conditions, corroborating the observed growth-supportive role of Fer in MDA-MB-231ΔFer xenografts progressing in mice under unrestricted diets [17]. However, a glucose-restricted diet further halted the progression of these tumors. The profound impeding effect of glucose depletion on the growth of Fer-lacking tumors was also manifested by the distorting effect of this metabolic burden on the tumors’ texture and integrity. This outcome could reflect the onset of death and/or growth attenuation in the Fer-deficient tumors.

Thus, the supportive role of Fer in sustaining metabolic plasticity of cancer cells gains profound importance in-vivo when a developing solid tumor outgrows its netting vasculature, thereby experiencing the onset of metabolic stress [54,55]. This also has important translational ramifications, envisaging a combined therapeutic approach consisting of targeting Fer with a selective inhibitor like E260 [15] and a deliberately given glucose-restrictive diet to the treated patients.

## 4. Materials and Methods

### 4.1. Cell Cultures

H358 cells (NCI-H358 (ExPASY Cellosaurus database Research Resource Identifier: CVCL_1559)) were kindly provided byYosef Yarden from the Weizmann Institute, Rehovot, Israel. MDA-MB-231 cells were purchased from the ATCC (ExPASY Cellosaurus database, Research Resource Identifier: CVCL_0062). The authentication of the parental H358 and MDA-MB-231 cells, and all their derived clones, was performed at the Genomic Center of Biomedical Core Facility, the Technion, Israel. All human cell lines were authenticated using STR profiling within the last three years, and all experiments were performed with mycoplasma-free cells. Cells were maintained in RPMI (H358 cells) Biological Industries-(01-100-1A, Beit-Haemek, Israel), or DMEM (MDA-MB-231 cells) (Gibco, Thermo Fisher Scientific, Waltham, MA, USA) supplemented with 10% fetal bovine serum (FBS) (Biological Industries, Beit-Haemek, Israel) and 5% PSN (Penicillin-Streptomycin-Nystatin) at 37 °C in a 5% CO_2_ atmosphere. DMEM glucose-free medium (Biological Industries-01-057-1A, Beit-Haemek, Israel) supplemented with 10% FBS (Biological Industries, Beit-Haemek, Israel), 5% PSN (Penicillin-Streptomycin-Nystatin) and 5% (0.3 g/L L-Glutamine (Biological Industries, Beit-Haemek, Israel) was used for growing the cells for seven days under glucose-deprivation conditions.

### 4.2. Generation of Stable CRSPR-Cas9 Knockout Clones

The *fer* gene alleles were knocked out in H358 cells and MDA-MB-231 cells using the Sigma CRISPR-Cas9 “paired nickases” system, according to the manufacturer’s instructions, using the cas9 D10A mutant [25], and a pair of gRNA (g4.1 RNA1: GCTTTGTCGTATCGTTCCTTGG, gRNA2: TTGCACAATCAGTATGTATTGG, Target ID: HSL0001703660 and HSR0001703662, respectively). Cells were transfected with plasmids using Lipofectamine 2000 (Invitrogen- 11668-019, Carlsbad, CA, USA) according to the manufacturer’s instructions. Cells expressing the GFP-fused cas9 were isolated by fluorescence-activated cell sorting (FACS) ARIA.

### 4.3. Western Blot Analysis

WB analysis was performed as previously described [16]. Thirty µg of whole cells protein lysates were resolved by 7.5% or 14% SDS-PAGE. Electro-blotted proteins were ATR-Thr1989 (CST-58014S), anti-ATR (CST-13934S), anti-CHK1-Ser345 (CST-2348S), anti-CHK1 (CST-2360S), anti-CHK2-Thr68 (CST-2661S), anti-CHK2 (CST-6334S), anti-H2AX-Ser139 (CST-9718), anti-H2AX (CST-7631S) and anti-CAD-Ser1859 (CST-12662S), all purchased from Cell Signaling. Anti-CAD (ab40800) and anti-ASS1 (ab124465) were purchased from Abcam. Anti-actin was purchased from Santa Cruz Biotechnology (Dallas, TX, USA). Anti-SH2antibodies were produced by our laboratory [16]. Immuno-absorbed antibodies were visualized using chemo-luminescence (Pierce, Rockford, IL, USA).

### 4.4. Immuno-Cytostaining

The immuno-cytostaining was performed as described previously [16]. The fixed cells were incubated overnight at 4 °C with anti-H_2_AX-Ser139 (Cell signaling- CST-9718, Danvers, MA, USA) as a primary antibody, diluted in blocking solution (1:200), and then with the secondary antibody at room temperature. The fluorescently stained cells were viewed with a Confocal microscope (Olympus-FV1000, Leica microsystems, Wetzlar, Germany).

### 4.5. Determination of Cell-Growth Curves

Cells were seeded in 24 well cell culture plates in 500 μL medium. Each sample was tested in duplicate, and cells were incubated at 37 °C in a 5% CO_2_ atmosphere. Growth medium was replaced once every 2–3 days; samples were trypsinized with 100 µL trypsin and then neutralized by adding 400 μL of medium containing 10% FBS. Cells were counted using a hemocytometer (Plastic Counting Chambers- REF 301890, Sigma-Aldrich, Rehovot, Israel). Each sample was counted twice and at a single timepoint for accuracy. In parallel, the cells were counted by using an automatic cell counter (Countess II, Life Technologies, Carlsbad, CA, USA) after the addition of Trypan blue to the sample.

### 4.6. Flow-Cytometry Cell-Cycle Analysis

Flow-cytometry cell-cycle analysis was performed as described previously [56]. Stain incorporation was measured by FACS Gallios, (FL3: excitation 488, emission 620), (Beckman Coulter, Indianapolis, IN, USA) and the results were evaluated using the FlowJo application. (Becton Dickinson, Franklin Lakes, NJ, USA).

### 4.7. Cell Death Analysis using Annexin/PI Staining

Cells were stained with Annexin V using the Annexin V-FITC Apoptosis Kit (BioVision Milpitas- k101-100, Milpitas, CA, USA,) according to the manufacturer’s instructions. Stain incorporation was measured by FACS Gallios (FITC: excitation 488, emission 530/30; PI: excitation 561, emission 610/20), and the results were evaluated using the FlowJo application (Becton Dickinson, Franklin Lakes, NJ, USA).

### 4.8. Measurement of ROS Levels

ROS levels were determined using the 2′,7′-dichlorofluorescin diacetate (DCFDA/H2DCFDA)–Cellular ROS Detection Assay Kit (Abcam- ab113851, Cambridge, UK) according to the manufacturer’s instructions. Stain incorporation was measured by FACS Gallios (excitation 488, emission 525/40 (FL1)), and the results were analyzed using the FlowJo application (Becton Dickinson, Franklin Lakes, NJ, USA).

### 4.9. Measurement of the Mitochondrial OXPHOS Complex I Activity

Activity of the mitochondrial OXPHOS complex I (NADH dehydrogenase) was determined using the Microplate Assay for Human Complex I Activity (Abcam, Complex I Enzyme Activity Microplate Assay kit (Colorimetric-, Cambridge, UK)), according to the manufacturer’s instructions.

### 4.10. Determining Mitochondrial Respiration Profile using “Mito-Stress” Analysis

Cells were seeded in 24 well cell culture plates (Seahorse XF24 FluxPak mini, 100867-100, Santa Clara, CA, USA), supplemented with 500 μL RPMI medium per well. After 48 h in culture, the growth media were changed to a DMEM glucose-free medium, and the cells were left in culture for an additional 24 h. The growth media of the cells were then changed to a Seahorse XF DMEM medium (103575-100, Santa Clara, CA, USA), supplied with an additional 0.3 g/L l-Glutamine (Biological Industries Beit-Haemek, Israel) and adjusted to pH = 7.4. Plates were incubated for 1 h at 37 °C in a CO_2_-free atmosphere. Mitochondrial respiration was measured by the Seahorse XF24 device after the addition of mitochondrial inhibitors at different time points, according to the manufacturer’s instructions (Seahorse XF Cell Mito Stress Test Kit, 103015-100, Santa Clara, CA, USA). To calculate the mitochondrial respiration capacity per cell, the cells were trypsinized and counted as above (Determination of cell-growth curves).

### 4.11. Measurement of the Mitochondrial Potential using TMRE Staining

Cells were stained with TMRE using the TMRE-Mitochondrial Membrane Potential Assay Kit (Abcam- ab113852, Cambridge, UK) according to the manufacturer’s instructions. One hundred nM TMRE was added for 20 min. Stain incorporation was measured by FACSAriaIII (FITC: excitation 545, emission 580), (Becton Dickinson, Franklin Lakes, NJ, USA) and the results were normalized per cell.

### 4.12. Determining NAD^+^ and Aspartate Levels

NAD^+^ and aspartate levels were determined using HPLC. Cells were seeded in 6 cm cell culture dishes and counted using an automatic cell counter for normalization. Cell lysis was performed using a Deproteinizing Sample Preparation Kit (Abcam - K808-200, Cambridge, UK) according to the manufacturer’s instructions. The cell lysates were filtered into HPLC vials and were loaded on a ReproSil 100 C18, 5 µm, 250 × 2 mm, r15.96.s 2502 column (Phenomenex, Aschaffenburg, Germany) for NAD^+^ quantification and on a Rezex ROA-Organic Acid H+ (8%) column (Phenomenex Aschaffenburg, Germany) for aspartate quantification. Injection volume was 10 µL and the running rate was 0.3 mL/min.

### 4.13. Quantification of Cellular ATP, CTP, TTP, Lys, Pro and Taurine Levels

Samples were trypsinized and then neutralized by adding a culture medium containing 10% FBS. Then, cells were centrifuged at 500 g for 5 min and washed in 0.5 mL PBS solution in 1.5 mL tubes. The cells were counted as described above and 1.5 million cells were centrifuged at 500 g for 5 min. The supernatants removed and cells were taken for LC-MS analysis at the Targeted Metabolomics Unit, Weizmann Institute of Science.

### 4.14. Animal Experiments

All animal experiments were performed according to the guidelines of the Institutional Animal Care and Use Committee. Immunocompromised “nude” mice (Harlan, Israel) were housed in a pathogen-free barrier environment, with up to five animals per cage with unlimited access to food and water and 12 h light/dark cycles. Experimental procedures involving laboratory mice were reviewed and approved by the Bar-Ilan University Animal Care Committee (Research approval numbers: 88-11-2018 and 10-02-2019) in compliance with the rules of the National Institute of Health, USA. Mice were fed with either an ad libitum (Altromin-1324 IRR, Lage, Germany) or a carbohydrate-deficient ketogenic diet (Altromin- C1073 glucose-deficient diet, Lage, Germany). The mice were transferred from an ad libitum diet to a carbohydrate-deficient ketogenic diet 3 days before tumor inoculation. The mice were kept on this diet throughout the experiment. Mice were subcutaneously inoculated with 4 × 10^6^ H358 or H358ΔFer cells, and the volume of the tumors was monitored every 3–4 days over a period of 5 weeks. Similarly, mice were subcutaneously inoculated with 2 × 10^6^ MDA-MB-231 or MDA-MB-231ΔFer cells and the volume of the tumors was monitored every 3–4 days over a period of 7 weeks.

Mice were sacrificed after 5 weeks of the xenograft experiments, as described above. The primary tumors were fixed in paraffin, cross-sectioned (6 µm sections) and stained with H&E for histopathological evaluation. The sections were inspected and photographed at the pathology unit of Tel Aviv Sourasky Medical Center.

### 4.15. Statistical Analysis

Statistical analysis was performed using a 2-tailed un-paired Student *t* test. Tumors’ development capacity was compared using a mixed linear regression approach. *p* < 0.05 was considered significant.

## Data Availability

All data presented in this manuscript will be made available upon request.

## Supplementary Materials

The following are available online at https://www.mdpi.com/1422-0067/22/7/3387/s1.
