# Supplementary material for: Loss of Fer Jeopardizes Metabolic Plasticity and Mitochondrial Homeostasis in Lung and Breast Carcinoma Cells
Source: Int J Mol Sci. 2021 Mar 25;22(7):3387. doi: 10.3390/ijms22073387 (PMC8037256; doi:10.3390/ijms22073387)
Supplement: Supplementary file 1 [file ijms-22-03387-s001.zip › Supplementary Material.pdf]

International Journal of
*Molecular Sciences*

MDPI

**Supplementary Material**

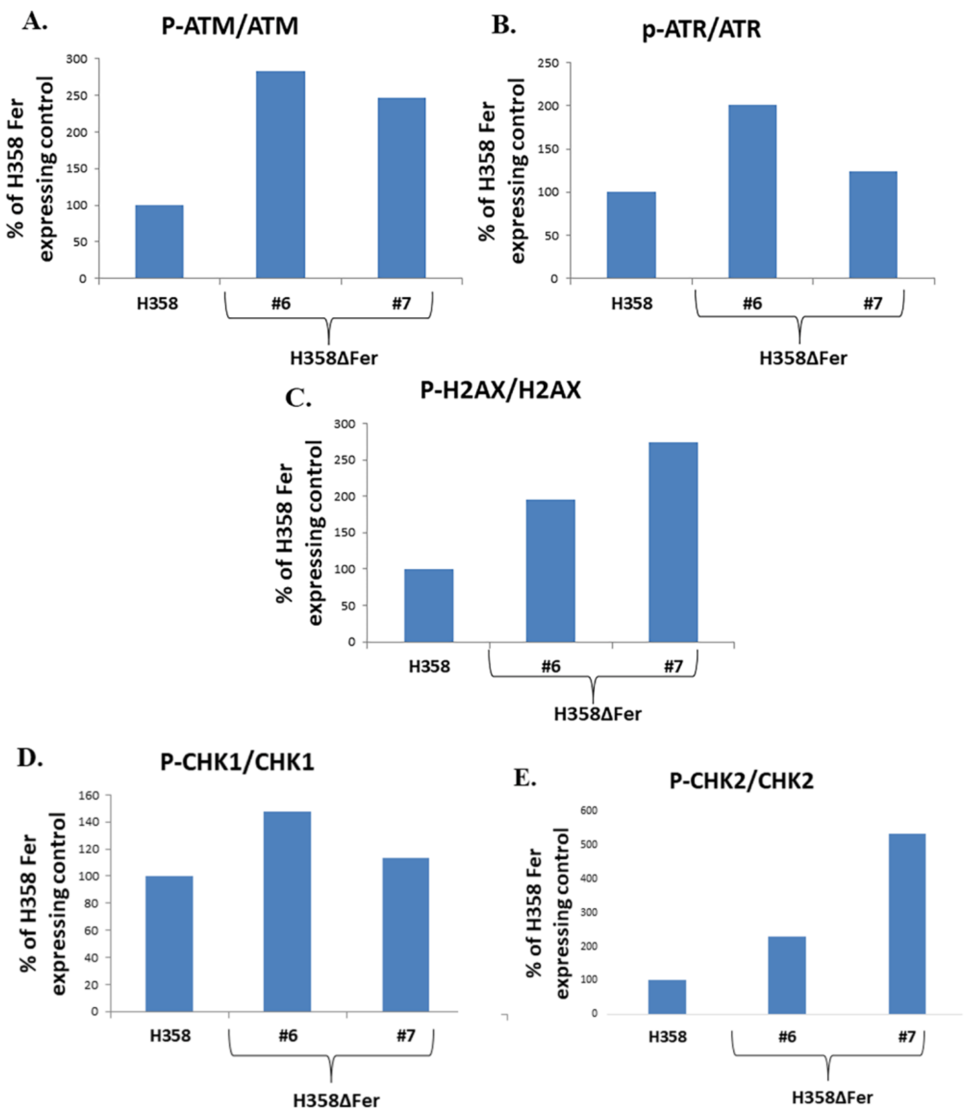


**Figure S1 . Presentation of phospho/pan ratio of the checked DDR proteins in H358 and H358ΔFer cells.** **[A]** P-ATM/ATM, **[B]** P-ATR/ATR , **[C]** P-H2AX/H2AX, **[D]** P-CHK1/CHK1, and **[E]** P-CHK2/CHK2. These results represent one out of three independent experiments which gave similar results.

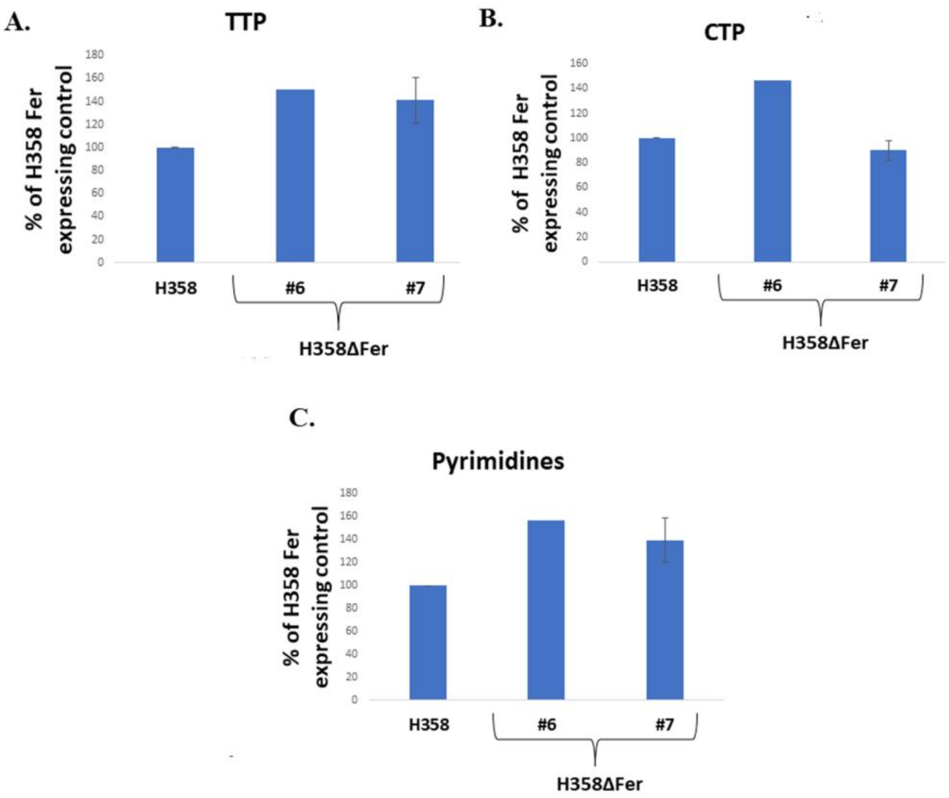


**Figure S2 ~~S1~~. similar pyrimidine levels in H358 and H358ΔFer cells**. **[A]** LC-MS analysis of TTP, and **[B]** CTP in H358 and H358ΔFer cells, grown in the absence of glucose. **[C]** Total TTP+CTP levels.

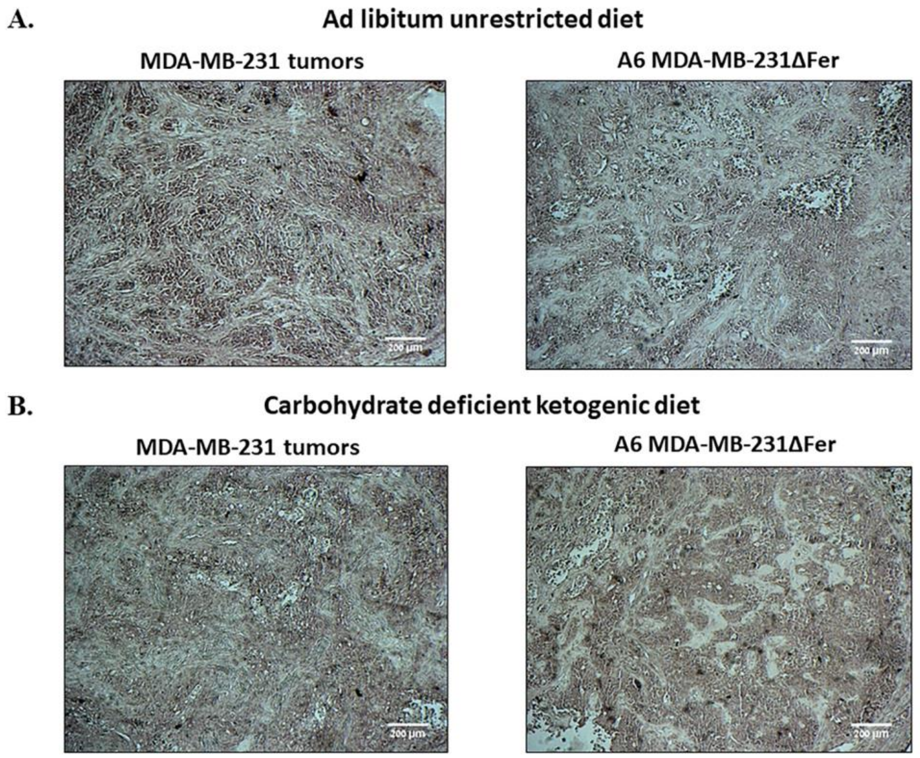


Figure S3 . Hematoxylin and eosin staining of MDA-MB-231, and MDA-MB-231ΔFer (clone A6) sections, derived from tumors in mice fed with [A] ad libitum un-restricted diet, or [B] a carbohydrate deficient ketogenic diet. Scale Bar: 200 µm.
