# Supplementary figures and images for: Loss of Fer Jeopardizes Metabolic Plasticity and Mitochondrial Homeostasis in Lung and Breast Carcinoma Cells
Source: Int J Mol Sci. 2021 Mar 25;22(7):3387. doi: 10.3390/ijms22073387 (PMC8037256; doi:10.3390/ijms22073387)

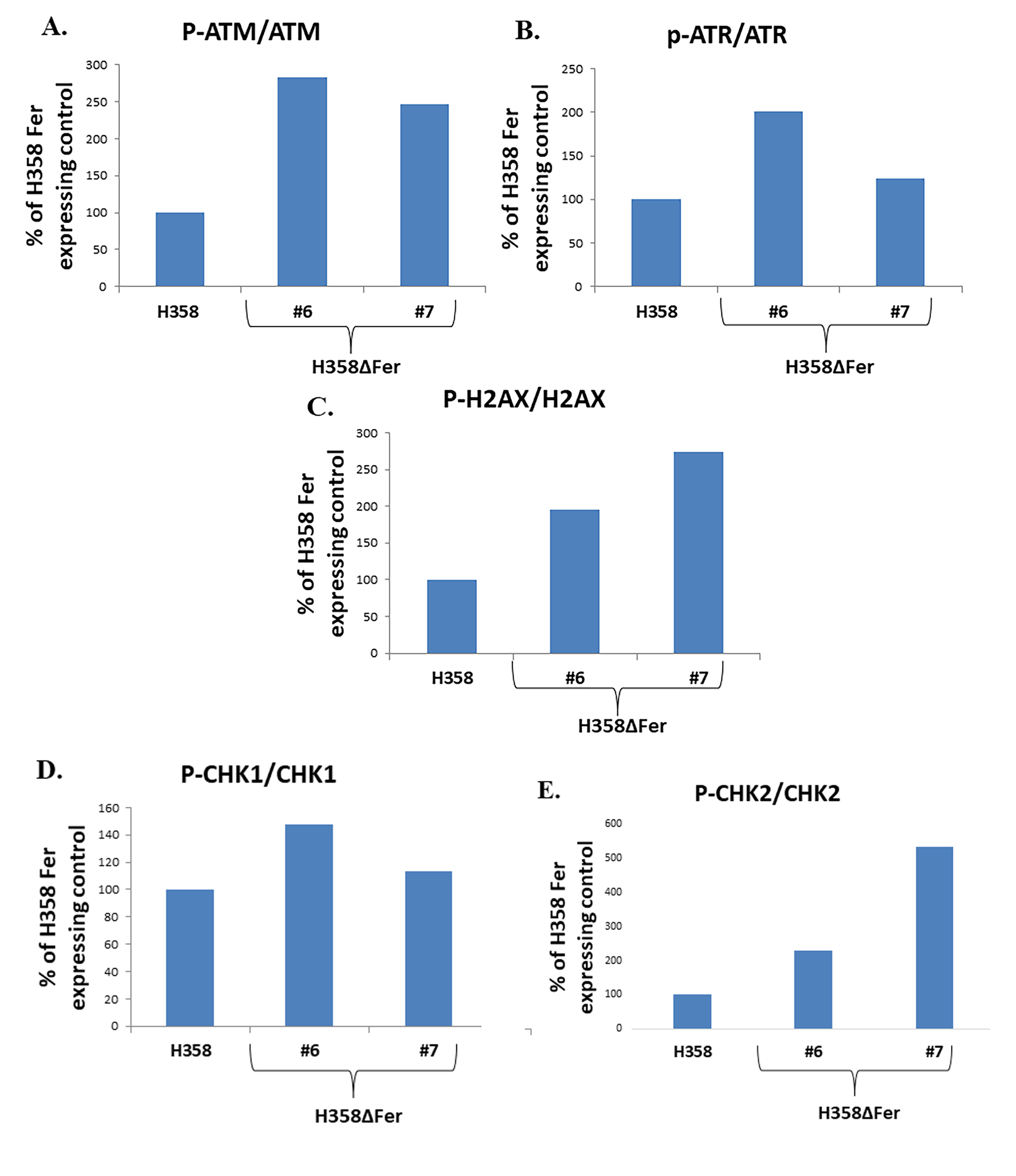

Supplement: Supplementary file 1 [file ijms-22-03387-s001.zip › Figure S1 17-03-21tif.tif]

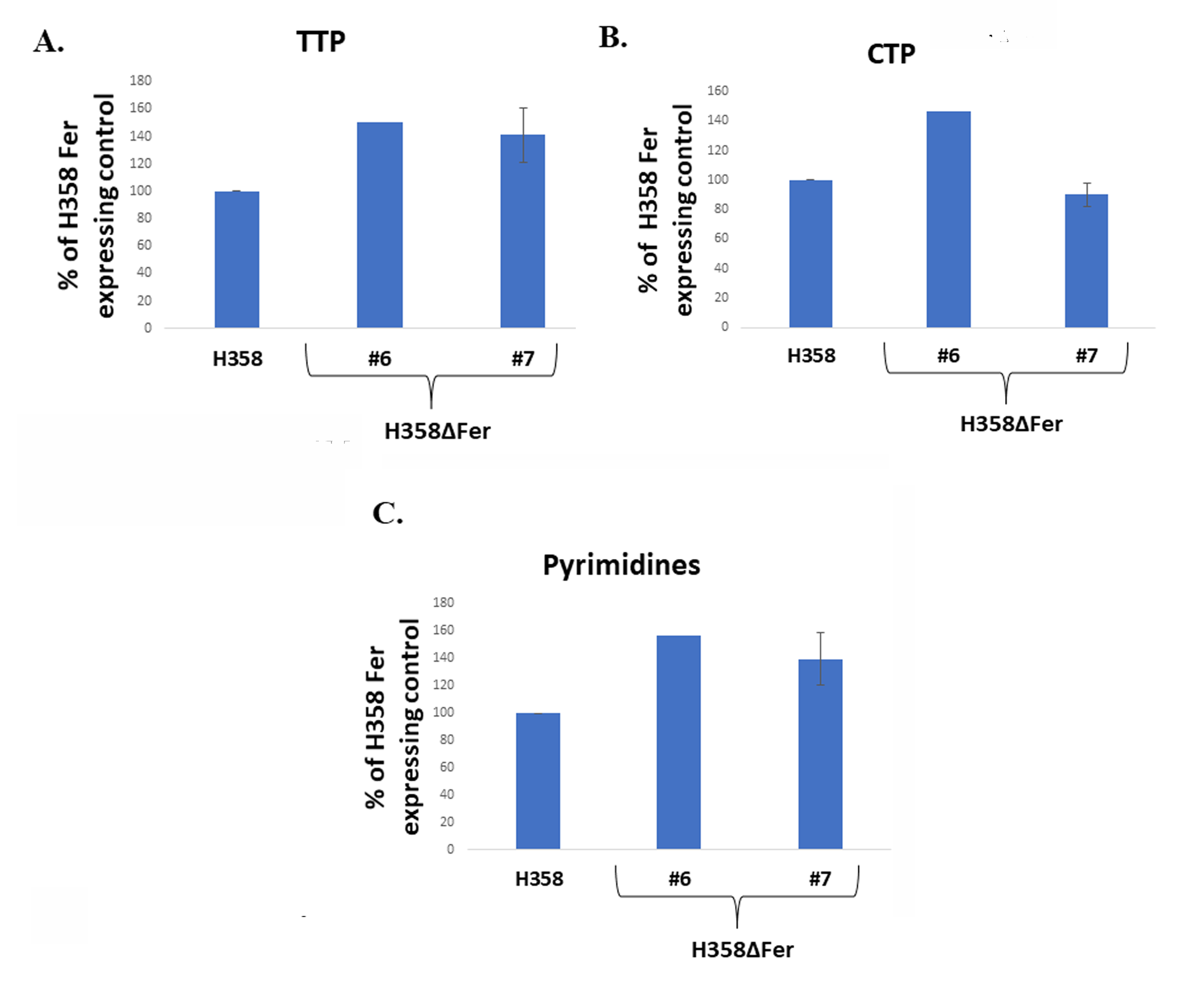

Supplement: Supplementary file 1 [file ijms-22-03387-s001.zip › Figure S2 17-03-21.tif]

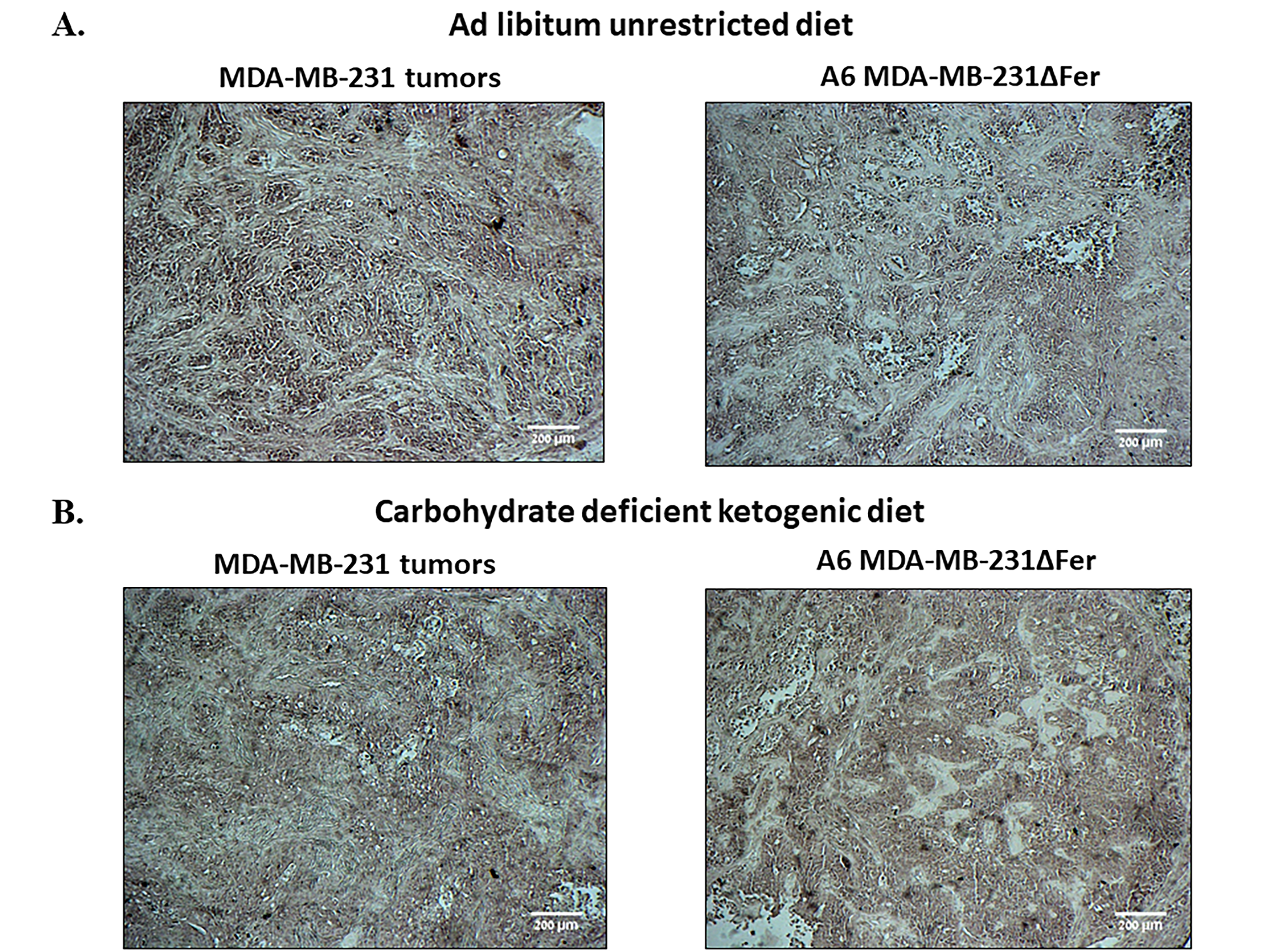

Supplement: Supplementary file 1 [file ijms-22-03387-s001.zip › Figure S3 17-03.tif]
